# Self-collected versus clinician-collected sampling for sexually transmitted infections: a systematic review and meta-analysis protocol

**DOI:** 10.1186/2046-4053-2-93

**Published:** 2013-10-10

**Authors:** Darlene Taylor, Carole Lunny, Tom Wong, Mark Gilbert, Neville Li, Richard Lester, Mel Krajden, Linda Hoang, Gina Ogilvie

**Affiliations:** 1 BC Centre for Disease Control, 655 West 12th Avenue, Vancouver, BC V5Z 4R4, Canada; 2 University of British Columbia, 2329 W Mall, Vancouver, BC V6T 1Z4, Canada; 3 Public Health Agency of Canada, 130 Colonnade Road, A.L. 6501H, Ottawa, ON K1A 0K9, Canada

**Keywords:** Self-collected, self-sampling, Physician-collected, Clinician-collected, Home sampling, Chlamydia, Gonorrhea, Human papillomavirus (HPV), STI, Diagnosis, Gold standard, Nucleic acid amplification tests

## Abstract

**Background:**

Three meta-analyses and one systematic review have been conducted on the question of whether self-collected specimens are as accurate as clinician-collected specimens for STI screening. However, these reviews predate 2007 and did not analyze rectal or pharyngeal collection sites. Currently, there is no consensus on which sampling method is the most effective for the diagnosis of genital chlamydia (CT), gonorrhea (GC) or human papillomavirus (HPV) infection. Our meta-analysis aims to be comprehensive in that it will examine the evidence of whether self-collected vaginal, urine, pharyngeal and rectal specimens provide as accurate a clinical diagnosis as clinician-collected samples (reference standard).

**Methods/Design:**

Inclusion and exclusion criteria: Eligible studies include both randomized and non-randomized controlled trials, pre- and post-test designs, and controlled observational studies. Search strategy: The databases that will be searched include the Cochrane Database of Systematic Reviews, Web of Science, Database of Abstracts of Reviews of Effects (DARE), EMBASE and PubMed/Medline. Data collection and analysis: Data will be abstracted independently by two reviewers using a standardized pre-tested data abstraction form. Heterogeneity will be assessed using the Q^2^ test. Sensitivity and specificity estimates with 95% confidence intervals as well as negative and positive likelihood ratios will be pooled and weighted using random effects meta-analysis, if appropriate. A hierarchical summary receiver operating characteristics curve for self-collected specimens will be generated.

**Discussion:**

This synthesis involves a meta-analysis of self-collected samples (urine, vaginal, pharyngeal and rectal swabs) versus clinician-collected samples for the diagnosis of CT, GC and HPV, the most prevalent STIs. Our systematic review will allow patients, clinicians and researchers to determine the diagnostic accuracy of specimens collected by patients compared to those collected by clinicians in the detection of chlamydia, gonorrhea and HPV.

## Background

Increasing rates of sexually transmitted infections (STIs) remain a growing concern worldwide [1-3]. Despite active and passive surveillance and multiple interventions aimed at increasing the number of cases found and treated, genital chlamydia (CT), gonorrhea (GC) and human papillomavirus (HPV) infections contribute to a large burden on health resources. Strategies to control STIs have included interventions aimed at improving access to screening among individuals who are at high risk of acquiring STIs. Current clinical screening methods for GC and CT involve the collection of cervical (endo- and ecto-), urethral, pharyngeal and rectal swabs. Urine and vaginal specimens are also collected for GC and CT. HPV testing using self-collected vaginal swabs is available but is not publicly funded, and, moreover, clinician-testing for HPV has not been adopted in Canada. Recent publications have identified a need for targeted site-specific screening of the pharynx, urethra and rectum to increase detection of cases, especially in individuals engaging in high risk activities such as men who have sex with men [4,5].

Self-collection of urine, vaginal, rectal and pharyngeal samples provides a different option for screening outside of the traditional clinical setting. Settings for self-collection of samples for STI testing can include community-based organizations, street outreach settings and the internet. However, more evidence is required to determine whether self-collected specimens are as accurate as clinician-collected specimens in terms of diagnostic accuracy for CT, GC and HPV, prior to recommending it as standard practice. The purpose of our systematic review and meta-analysis will be to examine whether a self-collected sample is as accurate as a clinical-collected sample for the diagnosis of CT, GC and HPV. Our specific research question is:

Are self-collected vaginal, urine, pharyngeal and rectal samples as accurate as clinician-collected samples for the diagnosis of CT, GC and HPV?

## Methods

### Inclusion and exclusion criteria

Eligible studies will include both randomized and non-randomized controlled trials, pre- and post-test designs, and controlled observational studies examining self-collected versus clinician-collected samples using the nucleic acid amplification test (NAAT) to detect GC, CT and HPV. All manuscripts must include a reference standard of a clinician-collected specimen. Manuscripts that only report a kappa statistic (measuring agreement between the self-collected sample and the clinician-collected sample) will also be reviewed. Table 1 delineates the eligibility guidelines for comparing sites for self-collected sample versus clinician-collected samples. Studies will be excluded when the time period between self- and clinician-collected samples is longer than three weeks because the disease status of the patient may have changed. Other reasons for exclusion are: (a) participants are known to be positive for disease before enrolment in the study, (b) combined test results (for example, self-collected combined urine and vaginal samples) or (c) more than 20% of included patients dropped out of the study.

**Table 1:** Eligibility guidelines for comparing sites for self-collected sample versus clinician-collected samples

		**Clinician-collected samples**			
		**Cervical**	**Rectal**	**Vaginal**	**Pharyngeal**
Self-collected samples	Urine	Eligible	Not	Eligible	Not
	Rectal	Not	Eligible	Not	Not
	Vaginal	Eligible	Not	Eligible	Not
	Pharyngeal	Not	Not	Not	Eligible

### Definition of reference standard

Our reference standard will be clinician-collected samples. Both self-collected and clinician-collected samples must have used a NAAT to be included. NAATs are highly sensitive and specific for the detection of CT and GC when using swabs from the genital tract or first-catch urine specimens [6]. The different NAATs for the detection of CT and GC include: PCR testing (Abbott LCx and Roche COBAS AMPLICOR™ (AC)), strand displacement amplification (Becton Dickinson ProbeTec™ ET), transcription-mediated amplification (Amptima, GenProbe Aptima Combo 2™ (AC2) and the Aptima *Neisseria gonorrhoeae* assay), and the ligase chain reaction (discontinued as of 2002) [6-8].

Dual assays that test for CT and GC, include AC2, Becton Dickinson ProbeTec™ CT/GC, AC and Abbott Realtime™ CT/NG assays [8-10]. The GenProbe PACE 2™, discontinued as of December 2012, and the Digene Corporation Hybrid Capture 2™ (HC2) are non-amplified probe tests [11]. HPV NAAT tests in this review will be classified as either HC2 or PCR [12,13]. Table 2 provides the sensitivity and specificity of clinician-collected specimens for GC and CT using NAAT assays.

**Table 2:** Sensitivity and specificity of reference standard (clinician-collected samples) for NAAT tests of gonorrhea and chlamydia

	**Vaginal swab**	**Rectal swab**	**Pharyngeal swab**	**Urine**
**Gonorrhea**				
Sensitivity	96.2 [13]	44-93 [14,15]	60–95 [14,15]	98.9 [16]
Specificity	99.3 [13]	99.5 [15]	78.9 [15]	99.3 [16]
**Chlamydia**				
Sensitivity	97.2 [13]	64–94 [14,15]	33–80 [14,15]	96.2 [16]
Specificity	95.2 [13]	100 [5]	100 [5]	98.1 [16]
**Gonorrhea/chlamydia combination**				
Sensitivity	97.2 [13]	100 [14,15]	95 [14,15]	100 [17]
Specificity	97–99.4 [13]	98 [14]	98 [14]	98.8 [17]

### Search strategy

Our search will include English and non-English databases. Our search will be limited to articles published since 1990 because of two factors: (a) the HC2 test is the most widely used HPV test worldwide and was first used in 1990 [14] and (b) the oldest currently available NAAT test reported in the literature for self-collected GC or CT was after 1990 [15]. Our search will be conducted on tests that ran between 1990 and June 2013. The databases that will be searched include the Cochrane Database of Systematic Reviews, Web of Science, Database of Abstracts of Reviews of Effects, EMBASE and PubMed/Medline. We have chosen not to register our protocol with PROSPERO.

### Search terms

MeSH headings, subject headings and keywords will be created using language that describes laboratory test performances for NAAT tests for GC, CT and HPV. The scope notes of the MeSH headings and subject headings and terms will be reviewed to identify additional terms, common usage and previous usage for terms being searched. Keywords in published journals will also be used. Search terms will include but will not be limited to: internet STI testing; sexually transmitted diseases; sexually transmitted infections; chlamydia; gonorrhea; human papillomavirus/HPV; screening; testing; reproductive health; adolescent health; non-clinic testing; urine testing; vaginal testing; pharyngeal testing; self-collected specimens; home sampling; test performance; test accuracy; PCR, polymerase chain reaction and nucleic acid amplification test sensitivity; specificity and diagnostic ratio. Boolean combinations will be created for more specific searches. Authors will also be asked to identify key articles that should be included in the review. The bibliographies of retrieved articles will also be manually searched as well as key journals such as *Sexually Transmitted Infections*, *Sexually Transmitted Diseases* and *Journal of Clinical Microbiology*.

### Data collection

Data will be abstracted independently by two reviewers using a standardized pre-tested form. Any disagreements between the reviewers will be resolved by a third reviewer. The following data elements will be extracted, and if appropriate, used for stratification if heterogeneity is found: true/false positive; true/false negative; sensitivity; specificity; reference (gold) standard used to compare test characteristics; NAAT platform used; specimen (urine, vaginal, pharyngeal or rectal); diagnostic odds ratio; positive likelihood ratio; negative likelihood ratio; kappa statistic; sex and age of participants; presence of symptoms (if a study includes results from both symptomatic and asymptomatic patients, both sets of results will be included); HIV status; date of study initiation and publication status (published or unpublished); country of study and author affiliation; number of specimens analyzed; number of tests per patient; any elements of blinding; location of self-collection (home, clinic and so on); community type (urban, rural); sex (women, men, transsexual); population type (stable, homeless); sexual orientation (gay, heterosexual, bisexual, transgendered); religion; clinician setting (outreach, primary care, referral); HPV type (high, low or both) and prevalence. If studies involve several self-sampling methods, the first method described will be used in the analysis. We consider any high-risk HPV positive test as standard; however, if the authors have not separated high- and low-risk HPV types, we used their combined data.

### Data analysis

The numbers of true positives, false negatives, false positives and true negatives will be extracted from each study, and test sensitivity and specificity as well as likelihood ratios will be calculated. Coupled forest plots for paired sensitivity and specificity for each study along with 95% confidence intervals will be created in RevMan 5.0 to detect publication bias, if present. A meta-analysis will be conducted according to the Cochrane Collaboration’s new recommended methodology for systematic reviews of Diagnostic Test Accuracy [16]. Hierarchical summary receiver operating characteristics (HSROC) analysis will be used, as described by Rutter and Gatsonis [17], as well as a bivariate random effects model for comparison purposes [18]. The HSROC model will be created, using SAS version 19, to provide a general framework for the meta-analysis and to allow for the calculation of the summary receiver operating characteristics (SROC) as well as the expected operating point on the curve and summary estimates of paired sensitivity and specificity (and positive/negative likelihood ratios). The HSROC will also be used to investigate heterogeneity between studies by taking into account both within- and between-study variability. Plots will not be fitted if there are four studies or less, as there will not be enough data to fit the hierarchical models with all five parameters by maximum likelihood. The Q statistic will be used to test for heterogeneity and publication bias will be examined. If heterogeneity is detected then the data will be examined to determine if stratification is appropriate. Studies will be stratified based on sub-grouping from data extraction. If heterogeneity is still evident pooled analyses will not be done and a descriptive summary will be reported.

### Quality

Validity will be assessed using the Quadas-2 (Quality Assessment of Diagnostic Accuracy Studies) checklist [19]. Sensitivity analyses will be conducted to investigate the effect of outliers on the summary estimates. The Cochrane Collaboration approach with risk of bias tables will be used to classify the studies as high, low or unclear [20]. The overall risk of bias for an individual study will be categorized as low if the risk of bias is low in all domains, unclear if the risk of bias is unclear in at least one domain without any high risk of bias domains or high if the risk of bias is high in at least one domain. The risk bias assessment will be performed by two reviewers independently and disagreements resolved by consensus.

## Discussion

### Scoping review

A search of the Cochrane Database of Systematic Reviews, Database of Abstracts of Reviews of Effects, EMBASE and PubMed/Medline using a review filter was conducted using the terms self-sampling and noninvasive sampling. One previous 2005 systematic review [21] evaluated 29 articles that reported sensitivity and specificity of urine-based NAATs for CT and GC with the specific aim of comparing self-collected urine samples with clinician-collected cervical and urethral samples. They reported congruent test accuracies between self-collection and physician-collected samples for CT, but lower sensitivity for GC in self-collected cervical samples compared to clinician-collected samples. This systematic review lacks information on pooled estimates of rectal or pharyngeal specimens. In addition, this review was published in 2005 and NAAT platforms have since changed significantly, and there are now platforms with greater test performance [7]. Dual assays, which test for both CT and GC, are now available [8,9]. Three other meta-analyses were conducted on the accuracy of self-collected vaginal specimens for diagnosing HPV compared to clinician-collected samples [22-24]; however, these reviews were conducted prior to 2007 and need updating. Moreover, these reviews did not include the rectum or pharyngeal anatomical sites as collection sites for specimens.

### Significance of the review

Increasing rates of STIs remain a growing concern in British Columbia, Canada, and worldwide. Rates of STIs continue to grow despite active and passive surveillance and multiple interventions aimed at increasing the number of cases found and treated. New interventions, such as self-collected sampling, are needed to identify STIs and stop the upward trend.

Reported rates of genital chlamydia (CT) have steadily increased in British Columbia since 1998, from 122/100,000 (1998) to 255/100,000 (2011), with the majority of cases being in females between the ages of 15 and 24 [2]. CT rates for young men in BC have also doubled since 1999 [1]. Similarly, the rate of gonorrhea infections has increased since 1998, from 529 new reported cases in 1998 to 1,573 new cases in 2011 [1,2]. The increased rates of GC and CT heighten concerns of increased rates of pelvic inflammatory disease, and vulnerability to acquiring HIV [2]. In addition, HPV is widespread in Canada and worldwide and persistent infection is correlated with an increased risk of cervical cancer [25,26].

The prevalence of HPV among women aged 13 to 86 is estimated to be 16.8% (with 10.7% being subtype 16 or 18 [27], which are the sub-types that cause 70% of cervical cancers [28]). The prevalence of HPV in men is less clear but cross-sectional studies of men in Vancouver found up to 27% of men have HPV subtype 16 or 18 [29,30].

### Barriers to accessing care

Uptake of STI testing remains a public health challenge, with particularly low testing rates among young men (age < 25) and gay, bisexual and other men who have sex with men (MSM) [30-37]. Stigma, shame, negative interactions with service providers, concerns about privacy and confidentiality, inaccessible locations, hours of operation, transportation costs and inconvenience have been identified as STI testing barriers [38-40]. In addition, not having a primary care provider [36] and fear of disclosing risky sexual behaviors to a care provider [37,41] have been shown to be factors that inhibit STI testing in some settings, especially for youths. Strategies to remove barriers to testing have been developed, such as internet-based testing using self-collected samples [42-44] or community-based screening for hard-to-reach populations [45,46].

### Models of internet-based self-sampling

A number of models for internet-based testing have been developed, all typically including an online risk self-assessment, test recommendations and specimen collection without requiring presentation to a clinic [47]. Self-collected specimens are taken in a community laboratory or at home [48-50]. The Clinical Prevention Services Division at the BC Centre for Disease Control is developing an internet-based testing program called *GetCheckedOnline*, aimed at decreasing barriers to STI and HIV testing with an emphasis on youth and MSM. Focus group discussions with youths, MSM and STI clinic clients revealed that they would use the internet-based testing service or recommend it to others [51]. The inclusion of self-collected specimens as part of this program is being considered [5]; however, knowing the accuracy of self-collection specimens compared to clinician-collected specimens for the diagnosis of STIs is required prior to adoption of this program. Ultimately, the goal of internet-based self-sampling programs would be to make screening more accessible, increase the number of people being tested, reduce onward transmission and provide timely treatment.

### Cost-effectiveness

Self-collected samples are also potentially more cost effective compared to clinician-collected samples. Organizing samples through the internet can reduce health service and delivery costs as interventions delivered over the internet are likely to cost less than treatments requiring frequent contact with health-care professionals [52]. Huang and colleagues [53] modelled an internet-based self-sampling chlamydia screening program for young women compared to a clinic-based screening and concluded that the internet-based screening strategy prevented 35.5 more cases of pelvic inflammatory disease and saved an additional US $41,000 in direct medical costs compared with the clinic-based screening strategy. A similar study found that self-obtained vaginal sampling for chlamydia detection was the least expensive and the most cost-effective method compared to clinician-collected or urine samples, preventing 17 more cases of pelvic inflammatory disease [54]. A third US study found that the lifetime cost of in-home self-collection for detection of high-risk HPV followed by in-clinic cytology triage was slightly lower than clinician-based screening [55].

The outcomes of this systematic review and meta-analysis are important in the detection of STI disease in hard-to-reach populations, in particular, those who may be unwilling to provide samples in traditional settings. The aim of exploring whether self-collected samples have comparable specificity and sensitively to samples collected in traditional settings has the potential to increase testing rates. Presumably, with higher testing levels, transmission could be reduced. Our systematic review will allow patients, clinicians and researchers to determine the diagnostic accuracy of specimens collected by patients compared to those collected by clinicians in the detection of chlamydia, gonorrhea, and HPV.

## Abbreviations

CT: Genital chlamydia; GC: Gonorrhea; HPV: Human papillomavirus; HSROC: Hierarchical summary receiver operating characteristics; MSM: Men who have sex with men; NAAT: Nucleic acid amplification test; PCR: Polymerase chain reaction; SROC: Summary receiver operating characteristics; STI: Sexually transmitted infection.

## Competing interests

The authors declare that they have no competing interests.

## Authors’ contributions

DT and NL drafted the manuscript. CL edited and finalized the manuscript. GO, RL and MG contributed to the research design. DT, CL, GO, MG, LH, MK and TW participated in writing the grant application and edited the manuscript. All authors read and approved the final manuscript.

## References

[B1] STI Prevention and Control STI/HIV Prevention and Control: 2009 Annual Surveillance Report 2009 Vancouver, BC: BC Centre for Disease Control 2009

[B2] Mayaud P McCormick D Interventions against sexually transmitted infections (STI) to prevent HIV infection Br Med Bull 2001 58 129 153 10.1093/bmb/58.1.129 11714628

[B3] BC Centre for Disease Control STI in British Columbia: 2011 Annual Surveillance Report 2011 Vancouver, BC: BC Centre for Disease Control http://www.bccdc.ca/util/about/annreport/default.htm

[B4] Dodge B Van Der Pol B Rosenberger JG Reece M Roth AM Herbenick D Fortenberry JD Field collection of rectal samples for sexually transmitted infection diagnostics among men who have sex with men Int J STD AIDS 2010 21 4 260 264 10.1258/ijsa.2009.009056 20378897

[B5] Gilbert M Salway Hottes T Chabot C Haag D Shoveller J Ogilvie G ‘There are a million scenarios to consider’: health care provider perspectives on internet-based testing for sexually transmitted infections, HIV and hepatitis C in British Columbia Poster for *STI & AIDS World Congress 2013* 2013 Vienna, Austria

[B6] Moncada J Donegan E Schachter J Evaluation of CDC-recommended approaches for confirmatory testing of positive *Neisseria gonorrhoeae* nucleic acid amplification test results J Clin Microbiol 2008 46 5 1614 1619 10.1128/JCM.02301-07 18322062 PMC2395091

[B7] Schachter J Hook EW Martin DH Willis D Fine P Fuller D Jordan J Janda WM Chernesky M Confirming positive results of nucleic acid amplification tests (NAATs) for *Chlamydia trachomatis*: all NAATs are not created equal J Clin Microbiol 2005 43 3 1372 1373 10.1128/JCM.43.3.1372-1373.2005 15750110 PMC1081269

[B8] Gaydos CA Quinn TC Willis D Weissfeld A Hook EW Martin DH Ferrero DV Schachter J Performance of the APTIMA combo 2 assay for detection of *Chlamydia trachomatis* and *Neisseria gonorrhoeae* in female urine and endocervical swab specimens J Clin Microbiol 2003 41 1 304 309 10.1128/JCM.41.1.304-309.2003 12517865 PMC149571

[B9] Gaydos CA Cartwright CP Colaninno P Welsch J Holden J Ho SY Webb EM Anderson C Bertuzis R Zhang L Miller T Leckie G Abravaya K Robinson J Performance of the Abbott RealTime CT/NG for detection of *Chlamydia trachomatis* and *Neisseria gonorrhoeae* J Clin Microbiol 2010 48 9 3236 3243 10.1128/JCM.01019-10 20668135 PMC2937681

[B10] Schachter J Which test is best for chlamydia? Curr Opin Infect Dis 1999 12 1 41 45 10.1097/00001432-199902000-00008 17035759

[B11] Darwin LH Cullen AP Crowe SR Modarress KJ Willis DE Payne WJ Evaluation of the hybrid capture 2 CT/GC DNA tests and the GenProbe PACE 2 tests from the same male urethral swab specimens Sex Transm Dis 2002 29 10 576 580 10.1097/00007435-200210000-00003 12370524

[B12] Eide ML Debaque H HPV detection methods and genotyping techniques in screening for cervical cancer Ann Pathol 2012 32 6 e15 e23 10.1016/j.annpat.2012.09.231 23244480

[B13] Poljak M Cuzick J Kocjan BJ Iftner T Dillner J Arbyn M Nucleic acid tests for the detection of alpha human papillomaviruses Vaccine 2012 30 Suppl 5 F100 F106 23199952 10.1016/j.vaccine.2012.04.105

[B14] Qiagen Hybrid Capture 2 Versus PCR: Are All HPV Tests Created Equal? 2013 http://www.qiagen.com/Knowledge-and-Support/Webinars/Molecular-Diagnostics/AttilaLorinczHC2/

[B15] Hobbs MM van der Pol B Totten P Gaydos CA Wald A Warren T Winer RL From the NIH: proceedings of a workshop on the importance of self-obtained vaginal specimens for detection of sexually transmitted infections Sex Transm Dis 2008 35 1 8 13 10.1097/OLQ.0b013e31815d968d 18157061 PMC3836284

[B16] Diagnostic Test Accuracy Working Group Handbook for DTA Reviews 2011 Birmingham UK: Cochrane Collaboration, Wiley Publications

[B17] Rutter CM Gatsonis CA A hierarchical regression approach to meta-analysis of diagnostic test accuracy evaluations Stat Med 2001 20 19 2865 2884 10.1002/sim.942 11568945

[B18] Dubourg J Berhouma M Cotton M Messerer M Meta-analysis of diagnostic test accuracy in neurosurgical practice Neurosurg Focus 2012 33 1 e5 10.3171/2012.5.FOCUS1295 22746237

[B19] Whiting PF Rutjes AWS Westwood ME Mallett S Deeks JJ Reitsma JB QUADAS-2: a revised tool for the quality assessment of diagnostic accuracy studies Ann Intern Med 2011 155 8 529 536 10.7326/0003-4819-155-8-201110180-00009 22007046

[B20] Higgins JPT Green S Cochrane Handbook for Systematic Reviews of Interventions Version 5.0.1 2008 Cambridge, UK: Cochrane Collaboration, Wiley Publications

[B21] Cook RL Hutchison SL Ostergaard L Braithwaite RS Ness RB Systematic review: noninvasive testing for *Chlamydia trachomatis* and *Neisseria gonorrhoeae* Ann Intern Med 2005 142 11 914 925 10.7326/0003-4819-142-11-200506070-00010 15941699

[B22] Ogilvie GS Patrick DM Schulzer M Sellors JW Petric M Chambers K White R FitzGerald JM Diagnostic accuracy of self collected vaginal specimens for human papillomavirus compared to clinician collected human papillomavirus specimens: a meta-analysis Sex Transm Infect 2005 81 3 207 212 10.1136/sti.2004.011858 15923286 PMC1744976

[B23] Petignat P Faltin DL Bruchim I Tramèr MR Franco EL Coutlée F Are self-collected samples comparable to physician-collected cervical specimens for human papillomavirus DNA testing? A systematic review and meta-analysis Gynecol Oncol 2007 105 2 530 535 10.1016/j.ygyno.2007.01.023 17335880

[B24] Stewart DE Gagliardi A Johnston M Howlett R Barata P Lewis N Oliver T Mai V HPV Guidelines Panel Self-collection guidelines panel. Self-collected samples for testing of oncogenic human papillomavirus: a systematic review J Obstet Gynaecol Can 2007 10 817 828 17915065 10.1016/s1701-2163(16)32636-6

[B25] Marra F Ogilvie G Colley L Kliewer E Marra CA Epidemiology and costs associated with genital warts in Canada Sex Transm Infect 2009 85 2 111 115 18981170 10.1136/sti.2008.030999

[B26] Cutts FT Franceschi S Goldie S Castellsague X de Sanjose S Garnett G Edmunds WJ Claeys P Goldenthal KL Harper DM Markowitz L Human papillomavirus and HPV vaccines: a review Bull World Health Organ 2007 85 9 719 726 10.2471/BLT.06.038414 18026629 PMC2636411

[B27] Moore RA Ogilvie G Fornika D Moravan V Brisson M Amirabbasi-Beik M Kollar A Burgess T Hsu R Towers L Lo J Matisic J Brooks-Wilson A Prevalence and type distribution of human papillomavirus in 5,000 British Columbia women – implications for vaccination Cancer Causes Control 2009 20 8 1387 1396 10.1007/s10552-009-9365-4 19475481 PMC2746887

[B28] Munoz N Bosch FX Castellsague X Diaz M de Sanjose S Hammouda D Shah KV Meijer CJ Against which human papillomavirus types shall we vaccinate and screen? The international perspective Int J Cancer 2004 111 2 278 285 10.1002/ijc.20244 15197783

[B29] Ogilvie GS Taylor DL Achen M Cook D Krajden M Self-collection of genital human papillomavirus specimens in heterosexual men Sex Transm Infect 2009 85 3 221 225 10.1136/sti.2008.033068 19066196

[B30] Rank C Gilbert M Kwag M Severini A van Niekerk D Ogunnaike-Cooke S Kropp R Gustafson R Moore D Ogilvie G the ManCount Survey Team Prevalence of Rectal Human Papilloma Virus Infection Among Men Who Have Sex With Men in Vancouver 2010 Vancouver, BC, Canada: Vancouver Coastal Health

[B31] Canada H Canada Communicable Disease Report 29–19 2003 Ottawa, ON: Scientific Publication and Multimedia Services

[B32] Wong T Singh A Mann J Hansen L McMahon S Gender differences in bacterial STIs in Canada BMC Womens Health 2004 4 Suppl 1 S26 10.1186/1472-6874-4-S1-S26 15345089 PMC2096668

[B33] Lewis DA Improving men’s sexual health: a challenge for today Sex Transm Infect 2004 80 6 423 424 15572605 10.1136/sti.2004.013540 PMC1744947

[B34] Moses S Elliott L Sexually transmitted diseases in Manitoba: evaluation of physician treatment practices, STD drug utilization, and compliance with screening and treatment guidelines Sex Transm Dis 2002 29 12 840 846 10.1097/00007435-200212000-00017 12466729

[B35] Berry SA Ghanem KG Page KR Thio CL Moore RD Gebo KA Gonorrhoea and chlamydia testing rates of HIV-infected men: low despite guidelines Sex Transm Infect 2010 86 6 481 484 10.1136/sti.2009.041541 20519251 PMC3066003

[B36] Mimiaga MJ Reisner SL Bland S Skeer M Cranston K Isenberg D Vega BA Mayer KH Health system and personal barriers resulting in decreased utilization of HIV and STD testing services among at-risk black men who have sex with men in Massachusetts AIDS Patient Care STDS 2009 23 10 825 835 10.1089/apc.2009.0086 19803696 PMC2859760

[B37] Shoveller J Johnson J Rosenberg M Greaves L Patrick DM Oliffe JL Knight R Youth’s experiences with STI testing in four communities in British Columbia Canada. Sex Transm Infect 2009 85 5 397 401 10.1136/sti.2008.035568 19508967

[B38] Fortenberry JD Health care seeking behaviors related to sexually transmitted diseases among adolescents Am J Public Health 1997 87 3 417 420 10.2105/AJPH.87.3.417 9096544 PMC1381015

[B39] Evans SJ Wright BL Goodbrand L Kilbreath JP Young J Teen sexuality. Reaching out in the malls Can J Public Health 2002 93 1 47 51 11925700 10.1007/BF03404417 PMC6980076

[B40] Goldenberg S Shoveller J Koehoorn M Ostry A Barriers to STI testing among youth in a Canadian oil and gas community Health Place 2008 14 4 718 729 10.1016/j.healthplace.2007.11.005 18171632

[B41] Klein JD Wilson KM Delivering quality care: adolescents’ discussion of health risks with their providers J Adolesc Health 2002 30 3 190 195 10.1016/S1054-139X(01)00342-1 11869926

[B42] Chai SJ Aumakhan B Barnes M Jett-Goheen M Quinn N Agreda P Whittle P Hogan T Jenkins WD Rietmeijer CA Gaydos CA Internet-based screening for sexually transmitted infections to reach nonclinic populations in the community: risk factors for infection in men Sex Transm Dis 2010 37 12 756 763 10.1097/OLQ.0b013e3181e3d771 20644498 PMC3187615

[B43] Prestage GP Hudson J Jin F Corrigan N Martin P Grulich AE McInnes D Testing for HIV and sexually transmissible infections within a mainly online sample of gay men who engage in group sex Sex Transm Infect 2009 85 1 70 74 10.1136/sti.2008.031120 19164606

[B44] Blas MM Alva IE Cabello R Garcia PJ Carcamo C Redmon M Kimball AM Ryan R Kurth AE Internet as a tool to access high-risk men who have sex with men from a resource-constrained setting: a study from Peru Sex Transm Infect 2007 83 7 567 570 10.1136/sti.2007.027276 17932128 PMC2598637

[B45] Auerswald CL Sugano E Ellen JM Klausner JD Street-based STD testing and treatment of homeless youth are feasible, acceptable and effective J Adolesc Health 2006 38 3 208 212 10.1016/j.jadohealth.2005.09.006 16488817

[B46] Kahn RH Moseley KE Thilges JN Johnson G Farley TA Community-based screening and treatment for STDs: results from a mobile clinic initiative Sex Transm Dis 2003 30 8 654 658 10.1097/01.OLQ.0000083892.66236.7A 12897689

[B47] Robinson TN Patrick K Eng TR Gustafson D An evidence-based approach to interactive health communication: a challenge to medicine in the information age. Science panel on interactive communication and health JAMA 1998 280 14 1264 1269 10.1001/jama.280.14.1264 9786378

[B48] Gaydos CA Dwyer K Barnes M Rizzo-Price PA Wood BJ Flemming T Hogan MT Internet-based screening for *Chlamydia trachomatis* to reach non-clinic populations with mailed self-administered vaginal swabs Sex Transm Dis 2006 33 7 451 457 10.1097/01.olq.0000200497.14326.fb 16652069

[B49] Gaydos CA Barnes M Aumakhan B Males will submit self-obtained penile swabs for the detection of *Chlamydia trachomatis* when recruited via the internet: acceptability and accuracy J Adolesc Health 2009 44 2 suppl S9

[B50] Woodruff A Levine DK Klausner JD Expanded STDtest.org Provides San Franciscans With Free Testing and Secure Online Results for five STDs 2008 National STD Conference: Chicago, IL

[B51] Hottes TS Farrell J Bondyra M Haag D Shoveller J Gilbert M Internet-based HIV and sexually transmitted infection testing in British Columbia, Canada: opinions and expectations of prospective clients J Med Internet Res 2012 14 2 e41 10.2196/jmir.1948 22394997 PMC3376521

[B52] Tate DF Finkelstein EA Khavjou O Gustafson A Cost effectiveness of internet interventions: review and recommendations Ann Behav Med 2009 38 1 40 45 10.1007/s12160-009-9131-6 19834778 PMC2772952

[B53] Huang W Gaydos CA Barnes MR Jett-Goheen M Blake DR Cost-effectiveness analysis of *Chlamydia trachomatis* screening via internet-based self-collected swabs compared with clinic-based sample collection Sex Transm Dis 2011 38 9 815 820 10.1097/OLQ.0b013e31821b0f50 21844736 PMC3156983

[B54] Blake DR Maldeis N Barnes MR Hardick A Quinn TC Gaydos CA Cost-effectiveness of screening strategies for *Chlamydia trachomatis* using cervical swabs, urine, and self-obtained vaginal swabs in a sexually transmitted disease clinic setting Sex Transm Dis 2008 35 7 649 655 10.1097/OLQ.0b013e31816ddb9a 18461013 PMC2711851

[B55] Balasubramanian A Kulasingam SL Baer A Hughes JP Myers ER Mao C Kiviat NB Koutsky LA Accuracy and cost-effectiveness of cervical cancer screening by high-risk human papillomavirus DNA testing of self-collected vaginal samples J Low Genit Tract Dis 2010 14 3 185 195 10.1097/LGT.0b013e3181cd6d36 20592553 PMC2898894

